# Lycorine Inhibits Influenza Virus Replication by Affecting Nascent Nucleoporin Nup93 Synthesis

**DOI:** 10.3390/ijms26115358

**Published:** 2025-06-03

**Authors:** Haiyan Yan, Huiqiang Wang, Kun Wang, Shuo Wu, Jiandong Jiang, Yuhuan Li

**Affiliations:** 1 CAMS Key Laboratory of Antiviral Drug Research, Beijing Key Laboratory of Technology and Application for Anti-Infective New Drugs Research and Development, NHC Key Laboratory of Biotechnology of Antibiotics, Institute of Medicinal Biotechnology, Chinese Academy of Medical Sciences and Peking Union Medical College, Beijing 100050, China; yan0495@163.com (H.Y.); hq_wangimb@163.com (H.W.); lexifree@163.com (K.W.); wushuoimb@126.com (S.W.); jiang.jdong@163.com (J.J.); 2 State Key Laboratory of Bioactive Substances and Functions of Natural Medicines, Institute of Medicinal Biotechnology, Chinese Academy of Medical Sciences and Peking Union Medical College, Beijing 100050, China

**Keywords:** influenza A virus, lycorine, antiviral activity, Nup93

## Abstract

The influenza A virus (IAV) is a major cause of recurrent seasonal epidemics and global pandemics, posing a significant threat to public health. Although lycorine has demonstrated broad-spectrum antiviral activity, its specific mechanisms of action against IAV remain incompletely understood. In this study, we characterized the potent inhibitory effects of lycorine on seasonal and drug-resistant IAV subtypes (H1N1/H3N2) as well as the influenza B virus, showing its ability to suppress viral mRNA, viral titers, and M2 protein expression across multiple cell lines. Time-of-addition and time-course assays revealed that lycorine exerts multiphasic interference, and the critical late stage of the IAV life cycle aroused our interest to study this further. Mechanistically, we discovered that lycorine specifically interferes with the de novo synthesis of nucleoporin Nup93, thereby disrupting the nuclear export of viral nucleoprotein (NP). These findings not only establish lycorine as a promising broad-spectrum anti-influenza candidate but also provide new insights for developing host-targeted antiviral strategies.

## 1. Introduction

The influenza A virus (IAV), a member of the Orthomyxoviridae family, harbors a negative-sense, single-stranded, segmented RNA genome. As a leading cause of human respiratory infections, IAV drives seasonal epidemics and sporadic pandemics, resulting in 3–5 million severe clinical cases annually [1,2]. Vulnerable populations, including infants, the elderly, and immunocompromised individuals face heightened risks of complications and mortality.

The replication cycle of influenza viruses is tightly regulated and highly dependent on host cellular machinery. Following entry via receptor-mediated endocytosis, viral ribonucleoprotein complexes (vRNPs) are released into the cytoplasm and subsequently imported into the nucleus—a critical step facilitated by the interaction between viral nucleoprotein (NP) and host importin-α/β [3]. Within the nucleus, viral RNA undergoes transcription and replication, producing progeny genomes that must then be exported back to the cytoplasm for virion assembly. This nuclear export process relies on the structural and regulatory functions of the nuclear pore complex (NPC) [4].

NPC is a part of nuclear machinery and orchestrates the bidirectional movement of macromolecules between the cytoplasm and the nucleus. This intricate structure is composed of approximately thirty distinct proteins, known as nucleoporins (Nups), which collectively form a basket-like framework [5]. Nups have been implicated in the IAV replication process, with notable examples such as Nup93, Nup98, Nup205, and Nup214 [6,7,8,9]. Notably, the FG domains of Nup214 have been identified as crucial for the interaction with the influenza NS2 protein, facilitating the export of vRNA [7]. Human nucleoporin 98 (hNup98) has been shown to engage in interactions with IAV NS2/nuclear export protein, and overexpression of its GLFG repeat domain has been observed to hinder the progression of viral propagation [8]. Nup93, a main component of the nuclear pore complex, has been hijacked by IAV in the nuclear export of viral RNA to ensure virus replication [9].

Lycorine, a bioactive isoquinoline alkaloid isolated from the bulb of the traditional medicinal plant *Lycoris radiata*, exhibits broad-spectrum antiviral activity against diverse pathogens, including SARS-CoV-2, human enterovirus A71 (EV-A71), Zika, dengue virus, and IAV H5N1 [10,11,12,13,14,15]. Its mechanisms span multiple stages of viral lifecycles: for instance, lycorine inhibits dengue virus replication by suppressing RNA-dependent RNA polymerase activity [13], while its antiviral effects on EV-A71 and Coxsackievirus A16 (CVA16) involve blocking virus-induced autophagy [11]. In the context of influenza, lycorine has demonstrated efficacy against H5N1 by inhibiting the nuclear-to-cytoplasmic export of the ribonucleoprotein (RNP) complex [15].

This study investigates the anti-influenza activity of lycorine against seasonal IAV strains, drug-resistant strains, and the influenza B virus (IBV) focusing on the late stages of the IAV replication cycle. We demonstrate that lycorine effectively suppresses the influenza A virus replication by suppressing de novo Nup93 synthesis without inducing degradation, thereby blocking vRNP nuclear export. Thus, lycorine has shown good anti-influenza virus activities by inhibiting nascent protein synthesis.

## 2. Results

### 2.1. Lycorine Inhibits the Replication of Influenza Virus

**Figure 1:**
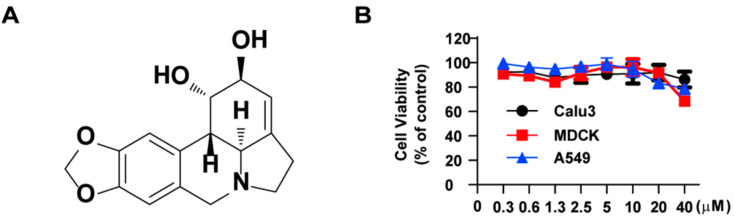
Chemical structure of lycorine and cytotoxicity assay. (**A**). Chemical structure of lycorine. (**B**). MDCK, A549, and Calu-3 cells were treated with lycorine at various concentrations. After 24 h, cytotoxicity was assessed using the CCK assay.

Prior to conducting antiviral research, the cytotoxicity of lycorine (the chemical structure showed in Figure 1A) was evaluated using the CCK assay in MDCK, Calu-3, and A549 cells to ensure that any observed antiviral effects were not mediated by cytotoxicity. The CC_50_ values were determined as 55.05 ± 6.09 μM in MDCK cells, 56.32 ± 10.77 μM in A549 cells, and 35.84 ± 6.83 μM in Calu3 cells. The results demonstrated that cell viability remained at approximately 90% after treatment with 10 µM lycorine for 24 h (Figure 1B). Based on these findings, a concentration range of 2–8 µM lycorine was selected for subsequent experiments.

**Figure 2:**
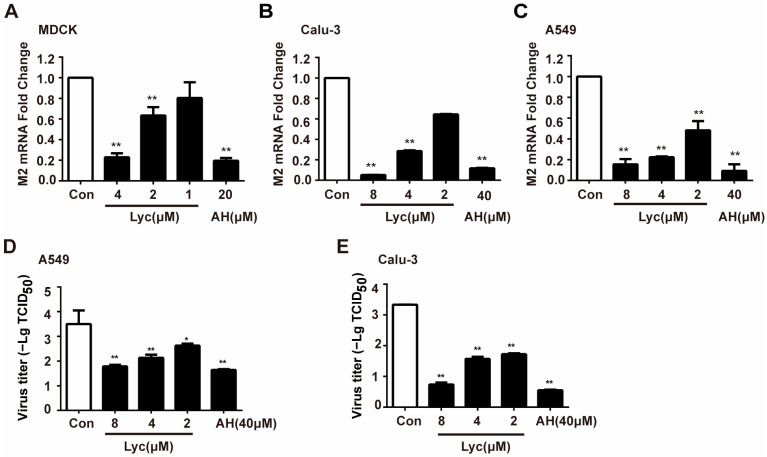
Quantification of anti-IAV effect of lycorine by qRT-PCR assay and viral titer determination. (**A**). MDCK, (**B**). Calu3, and (**C**). A549 cells were infected with IAV FM1 at multiplicities of infections (MOIs) of 0.002, 0.02, and 0.2, respectively, for 2 h and then treated with lycorine. AH was treated while infected. Total RNA was extracted, and the IAV M2 mRNA level was quantified using qRT-PCR. (**D**,**E**). A549 and Calu3 were infected with IAV at MOIs of 0.2 and 0.02, respectively, for 2 h and then added with lycorine. At 24 h post-infection, the cells were subjected to freeze–thaw cycles, and cell culture supernatants were collected. Viral titers were determined by cytopathic effects (CPE)-based TCID_50_ assay. Data are presented as means ± SD from triplicates of three independent experiments. * *p* < 0.05, ** *p* < 0.01 indicates a significant difference compared to the control (Con) group.

QRT-PCR was initially performed to determine whether lycorine affects viral mRNA levels. As shown in Figure 2A–C, lycorine significantly reduced IAV mRNA levels in MDCK, Calu-3, and A549 cells by 77%, 95%, and 85%, respectively, at a high concentration. Amantadine (AH), as a positive control drug, exhibits significant anti-influenza virus activity. Additionally, viral titers in A549 and Calu-3 cells dose-dependently decreased upon treatment with 8 μM lycorine, with an approximate 2-log reduction observed (Figure 2D,E). Collectively, these findings demonstrate the potent inhibitory effect of lycorine on influenza virus replication, with consistent efficacy across three cell lines and two viral subtypes (H1N1 and H3N2).

**Figure 3:**
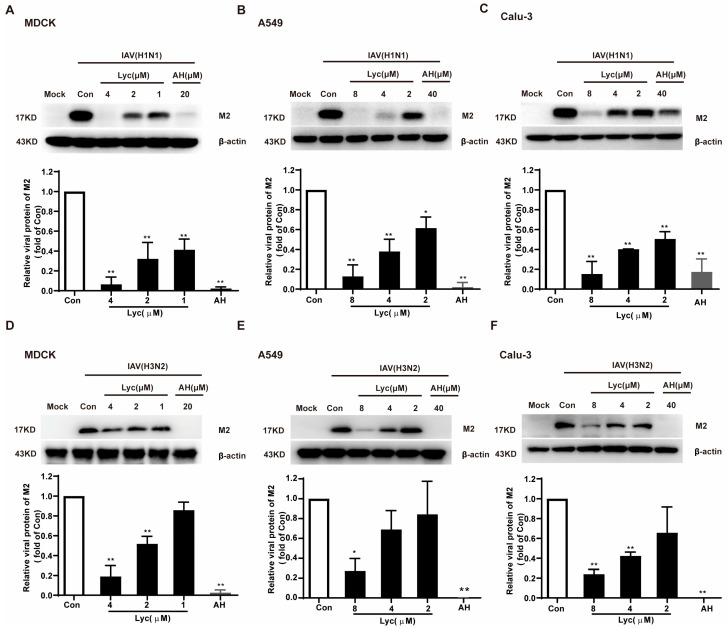
Lycorine inhibits H1N1 and H3N2 influenza virus replication at the protein level. MDCK, A549, and Calu-3 cells were infected with A/Fort Monmouth/1/1947 (FM1, H1N1) subtype (**A**–**C**) or A/Wuhan/359/95 (H3N2) subtype (**D**–**F**) at multiplicities of infection (MOIs) of 0.002 for MDCK, 0.2 for A549, and 0.02 for Calu-3. After 2 h of infection, the cells were incubated in the absence or presence of the indicated concentrations of lycorine for 24 h. AH was treated while infected. The protein levels of IAV M2 were analyzed by Western blot. The optical density ratio of the bands was quantified using the software ‘Image Lab 4.0’ (*n* = 2). * *p* < 0.05 and ** *p* < 0.01 indicate significant differences compared to the control (Con) group.

Next, the inhibitory effect of lycorine on IAV replication was assessed by measuring M2 protein expression levels in three cell lines infected with the H1N1 subtype. Lycorine significantly reduced M2 protein levels in MDCK, A549, and Calu-3 cells (Figure 3A–C), with inhibition percentages of 93.64%, 87.16%, and 84.77%, respectively, at a high concentration. Consistent results were obtained in a parallel study using the H3N2 subtype, with inhibition rates of 91.0%, 72.71%, and 76.0% observed in the same cell lines (Figure 3D–F).

**Figure 4:**
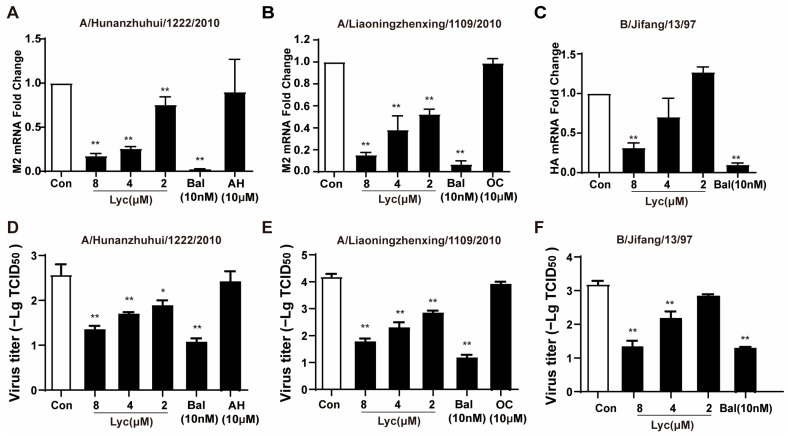
Effects of lycorine against drug-resistant strains of influenza virus and influenza B virus. (**A**). A/Hunanzhuhui/1222/2010, (**B**). A/Liaoningzhenxing/1109/2010, and (**C**). B/Jifang/13/97. MDCK cells were infected with IAV or IBV at MOI of 0.02 for 2 h and then treated with lycorine, Bal, and OC. AH was added while infected. Total RNA was extracted, and the IAV M2 or IBV HA mRNA level was quantified using qRT-PCR. (**D**–**F**). The same treatment was applied to the MDCK cells. At 24 h post-infection, the cells were subjected to freeze–thaw cycles, and cell culture supernatants were collected. Viral titers were determined by cytopathic effects (CPE)-based TCID_50_ assay. Data are presented as means ± SD from triplicates of three independent experiments. * *p* < 0.05 and ** *p* < 0.01 indicates a significant difference compared to the control (Con) group.

Drug resistance is one of the main reasons for the failure of clinical antiviral therapy, and previous studies have found that lycorine had significant antiviral effects on seasonal influenza viruses. Here, we investigated the effects of lycorine on the clinically isolated oseltamivir-resistant strain A/Liaoningzhenxing/1109/2010 (H1N1) and amantadine-resistant strain A/Hunanzhuhui/1222/2010 (H3N2). The results showed that lycorine exhibited a good inhibitory effect on the drug-resistant strains on MDCK cells (Figure 4A,B). In addition, we also performed an antiviral assay against the influenza B virus, and lycorine significantly inhibited the HA mRNA level of IBV (Figure 4C). The antiviral effect of the positive drug baloxavir (Bal) reached more than 90%; however, oseltamivir carboxylate (OC) and AH, as negative controls, showed no effect against resistant strains. In Figure 4D–F, lycorine also dose-dependently decreased viral titers in MDCK cells upon treatment with lycorine. These results suggest that lycorine has a broad-spectrum antiviral effect against the influenza virus.

### 2.2. Lycorine Affects the Multiple Stages of the Viral Replication Cycle Following Internalization

**Figure 5:**
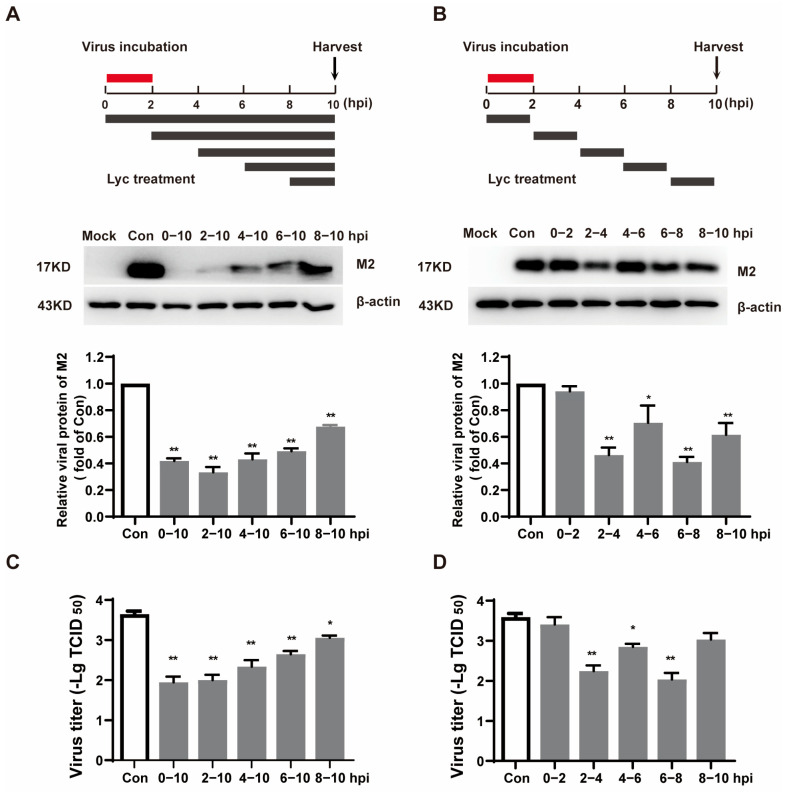
Lycorine targeted the post-entry stage of IAV infection. (**A**,**B**). A549 cells were infected with IAV (MOI = 5) at 0–2 h. Lycorine was treated at different time points above, and cells were collected at 10 hpi. IAV M2 was determined by Western blot. Software ‘Image Lab’ was used to analyze the optical density ratio of the bands (*n* = 2). (**C**,**D**). The same treatment was applied to the A549 cells. At 10 h post-infection, the cells were subjected to freeze–thaw cycles, and cell culture supernatants were collected. Viral titers were determined by cytopathic effects (CPE)-based TCID_50_ assay. * *p* < 0.05, ** *p* < 0.01 indicates significant difference from the Con group.

To identify the specific phase of the IAV life cycle targeted by lycorine, time-of-addition and time-course assays were conducted. In these experiments, cells were treated with lycorine at various time points either during or after infection, and the viral M2 protein levels were subsequently measured. As illustrated in Figure 5A, the lycorine treatment demonstrated significant antiviral activity at all tested time points from 0 to 10 h post-infection (hpi). Subsequent time-course analysis revealed that lycorine exhibited antiviral effects in four out of the five tested intervals: 2–4, 4–6, 6–8, and 8–10 hpi. Among these, the intervals of 2–4 hpi and 6–8 hpi showed the most pronounced antiviral activity (Figure 5B). The results of viral yield were consistent with those of the IAV M2 protein (Figure 5C,D). These results suggest that lycorine inhibits IAV replication by targeting multiple stages of the virus replication cycle, excluding the initial 2-h period during virus inoculation.

### 2.3. Lycorine Inhibits Nuclear Export of IAV NP

**Figure 6:**
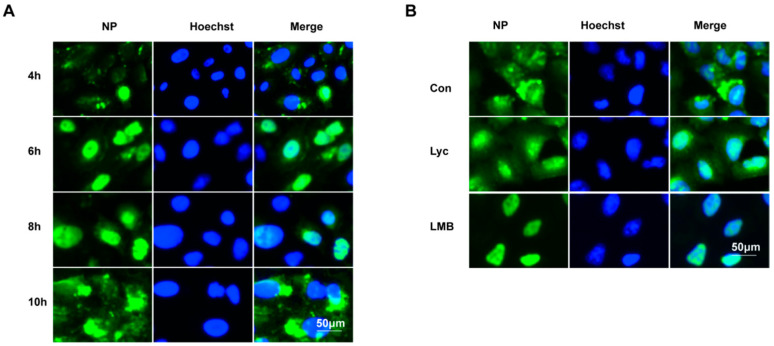
Lycorine inhibits nuclear export of IAV NP. (**A**). IAV NP localization in A549 cells (A/Fort Monmouth/1/1947(H1N1), 5 MOI) was conducted at 4 h, 6 h, 8 h, and 10 h post-infection. Cells were fixed followed by immunostaining the NP (green) and nucleus (blue). The images were acquired under a microscope with 20× lens. (**B**). A549 cells were infected with A/Fort Monmouth/1/1947(H1N1) at an MOI of 5 at 0–2 h and treated with 8 μM of lycorine and LMB 100 nM at 6–10 h. At 10 hpi, the cells were fixed and stained by IAV NP antibodies. The fluorescent cells were observed by fluorescence microscope (10 × 20).

A 2 h treatment with lycorine at 6–8 h post-infection (hpi) significantly inhibited IAV replication, prompting further investigation into its mechanism. Previous studies have demonstrated that lycorine can inhibit the export of the ribonucleoprotein (RNP) complex of the IAV H5N1 subtype from the nucleus [15]. To explore this effect, we performed immunofluorescence analysis to monitor the intracellular localization of the viral nucleoprotein (NP) in A549 cells following infection. NP is a major protein component of the genomic viral ribonucleoprotein (vRNP) and serves as a key marker for tracking vRNP dynamics. As shown in Figure 6A, a very small portion of the NP protein localizes in the cell nucleus at 4 hpi, and it is predominantly concentrated within the nucleus by 6 hpi. At 8 hpi, a portion of the NP protein begins to export from the nucleus, and by 10 hpi, the majority of the NP protein exhibits a diffused distribution throughout the cytoplasm.

Subsequently, the effect of lycorine on the localization of IAV NP was assessed using an immunofluorescence assay. Based on the dynamic data of NP localization obtained earlier, the time interval of 6–10 h post-infection (hpi) with lycorine treatment was selected to investigate the export of vRNP from the nucleus. Consistent with previous findings, lycorine treatment effectively inhibits vRNP nuclear export [15]. Notably, lycorine treatment during the 6–10 hpi interval achieves a comparable inhibitory effect with leptomycin B, a known inhibitor of protein export (Figure 6B). This result indicates that lycorine can effectively inhibit influenza virus replication by suppressing vRNP nuclear export, particularly when focusing on the nuclear export stage.

### 2.4. Lycorine Affects the Expression of Nup93

**Figure 7:**
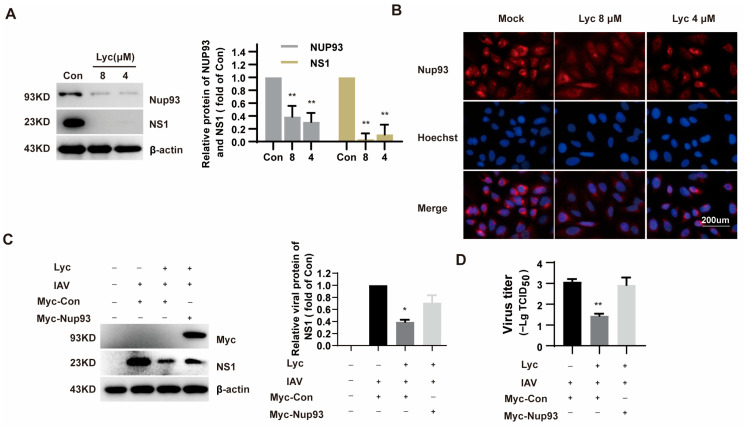
Lycorine affects the expression of Nup93. (**A**). A549 cells were treated with 8 µM and 4 µM lycorine with IAV infection at 0.2 MOI. Protein was harvested at 24 hpi and determined by Western blot. (**B**). A549 cells were treated with 8 μM and 4 μM of lycorine. Nup93 was detected by immunofluorescence. The cells were fixed and stained by Nup 93 antibodies. The fluorescent cells were observed by fluorescence microscope. (**C**). A549 cells transfected with Myc-Nup93 plasmid were infected with IAV at an MOI of 0.2 and treated with 4 μM of lycorine. Protein was harvested at 24 hpi, and viral NS1 was determined by Western blot. Software ‘Image Lab’ was used to analyze the optical density ratio of the bands (*n* = 2). (**D**). At 24 h post-infection, the cells were subjected to freeze–thaw cycles, and cell culture supernatants were collected. Virus titers were determined by cytopathic effects (CPE)-based TCID_50_ assay. * *p* < 0.05, ** *p* < 0.01 indicates significant difference from the Con group.

The nuclear pore complex (NPC) serves as a critical bridge facilitating the transport of proteins and RNAs between the nucleus and the cytoplasm in eukaryotic cells. However, the influenza virus can exploit the NPC to mediate the nucleocytoplasmic shuttling of viral proteins. From the above results, it is evident that lycorine blocks vRNP nuclear export. A previous study demonstrated that nucleoporin 93 (Nup93) plays a role in the nuclear export of influenza vRNP [9]. Interestingly, lycorine treatment was found to reduce the levels of Nup93 in H5N1-infected cells, as revealed by tandem mass tag-based quantitative proteomic analysis in the previous study [14]. As illustrated in Figure 7A, lycorine treatment markedly reduced the levels of both IAV NS1 and Nup93 proteins, aligning with earlier findings reported by Yang et al. [14]. The decrease in Nup93 protein levels was further confirmed through immunofluorescence experiments, which offered a more detailed visualization of this effect (Figure 7B). These results collectively reinforce the impact of lycorine on the observed protein reductions.

To further confirm that lycorine inhibits IAV replication by downregulating Nup93, we examined the effect of Nup93 overexpression on the antiviral activity of lycorine. Compared to the control vector (Myc-Con), overexpression of Nup93 partially reversed the inhibitory effect of lycorine on IAV NS1 protein levels as well as virus yield in A549 cells (Figure 7C,D). This result suggests that lycorine inhibits IAV replication, at least in part, through the downregulation of Nup93.

### 2.5. Lycorine Inhibits the Nascent Synthesis of Nup93 Protein

**Figure 8:**
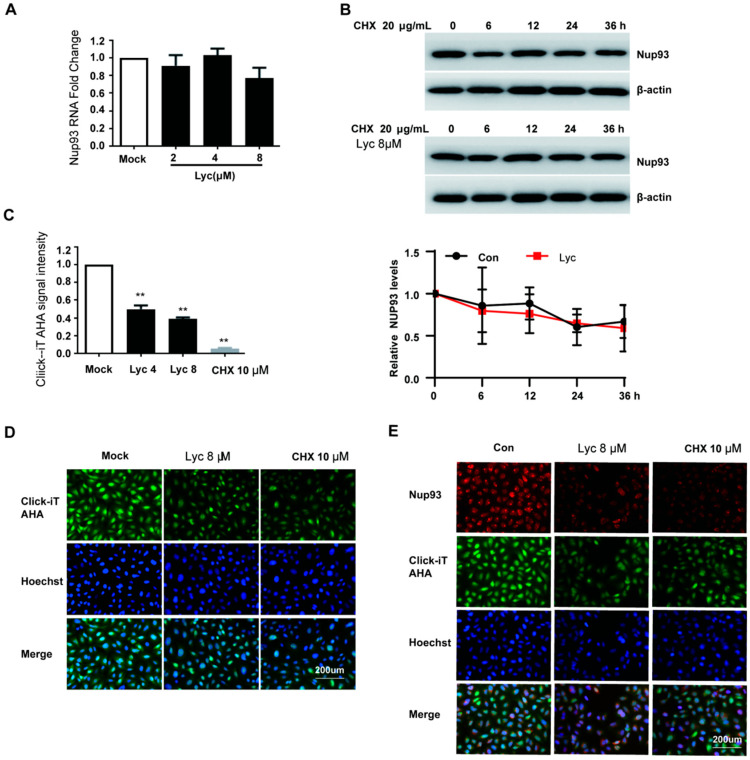
Lycorine inhibits the nascent synthesis of Nup93 protein. (**A**). A549 cells were treated with lycorine. Total RNA was extracted, and the Nup93 mRNA level was measured by quantitative RT-PCR. (**B**). A549 cells were incubated with cycloheximide (CHX, 20 μg/mL) and/or lycorine 8 μM for indicated times. Nup93 protein expression was analyzed by Western blot. Software ‘Image Lab’ was used to analyze the optical density ratio of the bands (*n* = 2). (**C**,**D**). A549 cells were treated in L-methionine-free media and 100 μM Click-iT AHA for 2 h with Lycorine (8 μM, 4 μM), or CHX (10 μM). (**E**). A549 cells were infected with IAV at an MOI of 0.2 and treated with lycorine (8 μM) or CHX (10 μM). Cells were washed, fixed, and permeabilized, and nascent protein synthesis was detected following a click reaction with Alexa Fluor 488 alkyne according to the manufacturer’s protocols. Imaging and quantitative analysis was performed using the fluorescence microscope and multimode plate reader platform. ** *p* < 0.01 indicates significant difference.

Previous observations demonstrated that lycorine treatment reduced Nup93 protein levels. To investigate the underlying mechanism, we first assessed the mRNA levels of Nup93 using qRT-PCR analysis. As shown in Figure 8A, lycorine treatment had no significant effect on Nup93 mRNA levels. Next, we evaluated the impact of lycorine on Nup93 protein stability using a cycloheximide (CHX) chase assay. The results revealed that lycorine treatment had no significant effect on the degradation kinetics of Nup93 (Figure 8B). Subsequently, nascent protein synthesis was measured using the Click-iT^®^ AHA Alexa Fluor^®^ 488 Protein Synthesis HCS Assay. The results revealed that lycorine treatment dose-dependently reduced total nascent protein synthesis in A549 cells (Figure 8C,D). Furthermore, lycorine significantly decreased the de novo synthesis of Nup93 protein in the context of IAV-infected A549 cells (Figure 8E). These findings indicate that lycorine exerts its antiviral activity against IAV by inhibiting Nup93 protein synthesis.

## 3. Discussion

As a natural alkaloid, lycorine has garnered significant attention in recent years due to its broad-spectrum antiviral activity against multiple viruses, including the IAV H5N1 subtype [10,11,12,13,14,16,17]. In the present study, we expanded the known antiviral spectrum of lycorine by demonstrating its efficacy against the seasonal influenza A virus subtypes (H1N1 and H3N2), oseltamivir- and amantadine-resistant clinically isolated strains, as well as the influenza B virus (IBV). Our findings reveal that lycorine significantly suppresses viral protein expression (M2, NS1), reduces viral mRNA levels, and decreases viral titers in H1N1 and H3N2 subtypes across diverse cell models. Importantly, lycorine also exhibits potent inhibitory effects against oseltamivir- and amantadine-resistant strains, as well as IBV, further confirming its broad-spectrum antiviral potential. Lycorine exhibits multiple pharmacological effects with diverse mechanisms of action. In terms of its antiviral activity, lycorine demonstrates varying mechanisms against different viruses. Lycorine acts as non-nucleoside RNA dependent RNA polymerase inhibitor that potently inhibits coronavirus, DENV, and ZIKV RNA synthesis [12,18,19]. Lycorine also targets host Hsc70 to inhibit HCV replication or to downregulate autophagy to inhibit EV-A71 and CVA16 replication [11,20].

For IAV, the previous study demonstrated that lycorine inhibits the H5N1 influenza virus by blocking viral ribonucleoprotein (vRNP) nuclear export [15], and this may be mediated by decreasing the Nup93 levels [14]. In the present study, we found that lycorine treatment during the 6–10 hpi interval can effectively inhibit vRNP nuclear export (Figure 6B). According to Yang’s study [14], lycorine significantly decreased the levels of Nup93 levels (Figure 7A,B). Subsequently, the antiviral effect of lycorine against IAV mediated by suppressing Nup93 levels was validated since the exogenous expression of Nup93 can attenuate the antiviral effect (Figure 7C). Lycorine has no effect on Nup93 mRNA levels and protein stability (Figure 8). Lycorine significantly inhibits the de novo synthesis of the Nup93 protein measured by the Click-iT^®^ AHA Alexa Fluor^®^ 488 Protein Synthesis HCS Assay (Figure 8C–E). This is consistent with its function of inhibiting translation [21,22]. This study reveals a profound molecular mechanism of “inhibiting de novo synthesis”, identifies the translation process as the action target, and makes advancements at the mechanistic level.

Notably, while our findings highlight the central role of Nup93 in lycorine’s antiviral activity, the NPC—comprising approximately 30 distinct nucleoporins—functions as a sophisticated molecular gateway regulating nucleocytoplasmic transport [23]. In the context of influenza virus replication, it has been discovered that Nup214 interacts with the NS2 protein of the influenza virus, and its knockout suppresses viral replication [7]. Human cellular protein nucleoporin hNup98 engages with viral NS2/nuclear export protein, with its GLFG sequence overexpression impeding viral propagation [8]. Nup85 associates with RNP subunits PB1/PB2 in an RNA-dependent manner during infection [24]. The protein synthesis inhibition of the lycorine effect on other nucleoporins needs to be investigated.

Time-of-addition and time-course assays revealed that lycorine acts as a multi-targeted molecular entity (Figure 5). In the present study, we focus on the late post-entry stage of IAV replication cycle. Other action stages such as 2–4 h treatment intervals also exhibited a significant antiviral effect which needs to be studied further.

In conclusion, our study identifies lycorine as a promising broad-spectrum anti-influenza agent with a unique mechanism of action involving the host nuclear pore protein Nup93. While we have elucidated a key aspect of its antiviral activity, further investigations are required to fully delineate the molecular mechanisms underlying lycorine’s inhibition of viral replication. These findings not only highlight lycorine’s therapeutic potential but also pave the way for the development of novel host-targeted antiviral strategies.

## 4. Materials and Methods

### 4.1. Cell Culture and Virus

Madin–Darby Canine Kidney (MDCK) cells were obtained from the American Type Culture Collection and cultured in a minimum essential medium (MEM) supplemented with 10% fetal bovine serum (FBS), 1% Penicillin-Streptomycin (PS, 10,000 U/mL), and an extra 1% nonessential amino acid solution. Human lung adenocarcinoma cell line cells (Calu-3) and Human lung adenocarcinoma epithelial cells (A549) were purchased from the National Infrastructure of Cell Line Resource. Calu-3 were grown at 37 °C in MEM supplemented with 10% FBS and 1% PS. A549 were maintained in an F-12K medium supplemented with 10% FBS and 1% PS.

The influenza virus A/Fort Monmouth/1/1947 (FM1, H1N1) was purchased from the America Type Culture Collection (ATCC, VR-97^TM^). A/Wuhan/359/95 (H3N2), A/Hunanzhuhui/1222/2010 (H3N2, adamantanes resistant clinical isolate), A/Liaoningzhenxing/1109/2010 (H1N1, oseltamivir-resistant clinical isolate), and B/Jifang/13/97 were kindly provided by Professor Yuelong Shu at the Institute for Viral Diseases Control and Prevention, the Chinese Center for Disease Control and Prevention. Unless otherwise noted, the influenza virus A/Fort Monmouth/1/1947 was used in the experiments.

### 4.2. Compounds

Lycorine hydrochloride (Lyc), Leptomycin B (LMB), Baloxavir (Bal), Oseltamivir (OC), and Amantadine (AH) were purchased from MedChemExpress (Monmouth Junction, NJ, USA). Cycloheximide (CHX) was purchased from Sigma Aldrich (St. Louis, MO, USA). All the compounds were dissolved in dimethyl sulfoxide (DMSO).

### 4.3. Cytotoxicity Assay

The cytotoxic effect of lycorine on growing MDCK, Calu-3, and A549 cells were estimated by the Cell Counting Kit (CCK) assay, according to the manufacturer’s instructions (TransGen Biotech, Beijing, China).

### 4.4. Anti-IAV Assay

MDCK (4.5 × 10^5^ cells/well), A549 (1.5 × 10^5^ cells/well), and Calu3 (1.5 × 10^5^ cells/well) cells were seeded in 12-well culture plates. Following overnight culturing, cells were mock-infected or infected with IAV for 2 h. Then, they were treated with different concentrations of lycorine or positive compounds for 24 h. The cells were harvested for Western blot assay, qRT-PCR assay, and viral titers.

### 4.5. Western Blot

The cellular proteins were extracted using M-PER Mammalian Protein Extraction Reagent (Thermo Fisher Scientific, Waltham, MA, USA) with a halt protease inhibitor single-use cocktail at 4 °C for 30 min. Cellular debris was removed by centrifuge at 12,000× *g* for 15 min at 4 °C, and the lysates were mixed with loading buffer.

Protein extracts (approximately 10 µg) were subjected to sodium dodecyl sulfate-polyacrylamide gel (SDS-PAGE). Western blot was performed by the dilution of the primary antibodies: matrix-2 (M2), NS1, Nup93 (Santa Cruz, CA, USA), NP (Abcam, Cambridgeshire, UK), and β-actin, Myc (Cell Signaling Technology, Danvers, MA, USA). After washing, blots were incubated with a goat anti-rabbit or anti-mouse HRP-conjugated secondary antibody (Cell Signaling Technology, Danvers, MA, USA). The signal was detected with Pierce Enhanced chemiluminescent HRP substrate (Thermo Fisher Scientific, Waltham, MA, USA).

### 4.6. Real-Time Quantitative PCR Analysis

The total RNA was isolated by using RNeasy Mini Kit (Qiagen, Hilden, Germany) according to the manufacturer’s instructions and analyzed with the TransScript^®^ II Green One-Step qRT-PCR SuperMix kit (TransGen Biotech, Beijing, China). Relative ratios were determined based on the 2-ΔΔCT method. Primers were synthesized by Sangon (Shanghai, China). The specific primers used for the one-step qRT-PCR assay are listed in Table 1.

### 4.7. Virus Titer Determination

The Calu-3 and A549 cells were harvested at 24 hpi. After three freeze–thaw cycles, the virus titers were determined in MDCK cells via the cytopathic effect (CPE) assay. Confluent monolayers of MDCK cells were infected with a 10-fold serially diluted supernatant in 96-well plates, and the cytopathic effect of cells was observed at 48 hpi. TCID_50_ was calculated by Reed & Muench method.

### 4.8. Time-of-Addition Assay and Time-Course Assay

Time-of-addition and time-course experiments were used to analyze the viral replication step targeted by lycorine. Briefly, A549 cells were inoculated with 5 MOI of IAV at 37 °C for 2 h and treated with lycorine at the concentration of 8 µM at different time intervals. After 10 hpi, the cellular proteins were extracted, and the viral M2 protein was detected by a Western blot assay.

### 4.9. Immunofluorescence

For immunofluorescence analysis, A549 cells were fixed at times indicated by 4% paraformaldehyde for 15 min, permeabilized in 0.5% Triton X-100 for 15 min, and blocked with PBS containing 1% BSA for 1 h at room temperature. Cells were immunolabeled with Nup93 antibody (Santa Cruz, CA, USA) or IAV NP antibody (Abcam, UK) overnight at 4 °C and 1 h with Alexa-Fluor 488 conjugated donkey anti-mouse IgG secondary antibodies (TransGen Biotech, China). After washing with PBST, the nucleus was stained with Hoechst 33342 (Beyotime Biotechnology, China), and images were taken with a fluorescence microscope (ZEISS, X-Cite 120Q, Oberkochen, Germany).

### 4.10. Nascent Protein Synthesis Assay

The Nup93 nascent protein synthesis assay was determined using the Click iT l-azidohomoalanine (AHA) Alexa Fluor 488 Protein Synthesis HCS Assay kit (Invitrogen, Waltham, MA, USA) with a slight modification. A549 cells were treated in L-methionine-free media and 100 μM Click-iT AHA for 2 h with Lycorine (8 μM or 4 μM) or cycloheximide (10 μM). Cells were washed, fixed, permeabilized, and nascent protein synthesis was detected following a click reaction with Alexa Fluor 488 alkyne according to the manufacturers’ protocols. Imaging and analysis were performed using the fluorescence microscope (ZEISS, X-Cite 120Q, GER, Oberkochen, Germany) and a multimode plate reader platform (Perkin Elmer, Waltham, MA, USA).

### 4.11. Statistical Analysis

Statistical analysis was performed with GraphPad Prism 8.0. All results are expressed as mean ± SD. The difference between groups was compared by one-way ANOVA, followed by LSD or Dunnett’s multiple comparison test. The * *p* < 0.05 and ** *p* < 0.01 were considered to be significant.

## Figures and Tables

**Table 1:** Primers used in qRT-PCR assay.

Genes	Sequence (5′-3′)
Influenza *M2*-F	GACCRATCCTGTCACCTCTGAC
Influenza *M2*-R	GGGCATTYTGGACAAAKCGTCTACG
Influenza *HA*-F	CAAACAGAAGACGGAGGACTACCAC
Influenza *HA*-R	ATTACCTTGCTCCTGCCACTTGC
*GAPDH*-F(H)	GGTGGTCTCCTCTGACTTCAACA
*GAPDH*-R(H)	GTTGCTGTAGCCAAATTCGTTGT
*Gapdh*-F(D)	AGTCAAGGCTGAGAACGGGAAACT
*Gapdh*-R(D)	TCCACAACATACTCAGCACCAGCA

Note: H: homo; D: dog.

## Data Availability

The datasets generated during the present study are available from the corresponding author upon reasonable request.
